# Genome Sequence of the Solvent-Producing Clostridium beijerinckii Strain 59B, Isolated from Staffordshire Garden Soil

**DOI:** 10.1128/genomeA.00108-15

**Published:** 2015-03-19

**Authors:** Gareth T. Little, Klaus Winzer, Nigel P. Minton

**Affiliations:** The Clostridia Research Group, BBSRC/EPSRC Synthetic Biology Research Centre, School of Life Sciences, Centre for Biomolecular Sciences, University of Nottingham, Nottingham, United Kingdom

## Abstract

The genome sequence of the solvent-producing, spore-forming, saccharolytic, mesophilic bacterium *Clostridium beijerinckii* strain 59B, isolated from Staffordshire garden soil, was obtained via a combination of sequencing with the 454 and Illumina platforms. This information will allow for metabolic engineering of a potentially industrially useful strain.

## GENOME ANNOUNCEMENT

Increasing awareness of the need for sustainable sources of fuels and chemicals has resulted in the need for a larger pool of potentially industrially useful organisms. During the first half of the 20th century, bacteria of the genus Clostridium were used for the production of solvents from crops (1). Environmental samples obtained from a range of locations were screened for solvent and spore-forming microorganisms. A promising candidate organism identified by 16S rRNA gene sequencing as a *Clostridium beijerinckii*, isolated from garden soil in Staffordshire, United Kingdom, was selected for further study.

Genomic DNA was extracted after lysis by incubation with lysozyme, proteinase K, and SDS buffer, with phenol-chloroform-isoamyl alcohol (2). A mate-paired library containing a 3-kb insert was prepared from the genomic DNA. This library was sequenced using one channel on an Illumina HiSeq2000 instrument by GATC Biotech (Germany), from which 109,185,408 reads were obtained, all of which were 51 bp long. In addition, the genome was sequenced using a quarter plate on the Roche 454-GS FLX platform, from which 215,290 sequences were obtained.

Genomics Workbench 6.5 (CLC bio, Denmark) was used for assembly of both sets of data, resulting in 106 contigs. GapFiller was used for closing of some of these gaps using the Illumina data (3). To facilitate the correct alignment of the contigs, an optical map using the restriction enzyme *NcoI*, which produced 540 fragments and a total length of 6,447,845 (mean fragment length 11.9 kbp), was prepared (OpGen, USA). Contigs were aligned using the software MapSolver v.3.2.0. Any gaps remaining between contigs were filled with stretches of the ambiguous base character “N” corresponding to the estimated size indicated by the optical map. The final assembled sequence length is 6,455,117 bp with an average coverage of 800×.

The nucleotide sequence was annotated using the PROKKA prokaryotic genome annotation pipeline (4) followed by manual corrections. The number of annotated features are as follows: coding sequence (CDS), 5,592; gene, 5,717; miscellaneous binding, 58; miscellaneous feature, 5; signal peptide, 330; noncoding RNA (ncRNA), 23; rRNA, 17; and tRNA, 69. Strain 59B showed 100% 16S rRNA sequence similarity with strains *Clostridium beijerinckii* ATCC 35702 and *Clostridium beijerinckii* NCIMB 8052. Analysis of the sequence with the CAZYmes Analysis Toolkit identified 946 genes with carbohydrate-binding activity (5). No cellulosome component-encoding genes were annotated in this genome sequence. Seven candidate clustered regularly interspaced short palindromic repeat (CRISPR) sequences were identified (6).

Having the genome sequence of this organism will allow for precise metabolic engineering to improve the solvent production characteristics of this strain. The annotated sequence will also further the understanding of other solvent-producing Clostridium sp.

### Nucleotide sequence accession number.

The genome sequence has been deposited in GenBank with the accession number CP010086.
